# Conformational transition of the *Ixodes ricinus* salivary serpin Iripin-4

**DOI:** 10.1107/S2059798323002322

**Published:** 2023-04-24

**Authors:** Barbora Kascakova, Jan Kotal, Petra Havlickova, Vera Vopatkova, Tatyana Prudnikova, Pavel Grinkevich, Michal Kuty, Jindrich Chmelar, Ivana Kuta Smatanova

**Affiliations:** a Department of Chemistry, University of South Bohemia in České Budějovice, 370 05 České Budějovice, Czech Republic; b Department of Medical Biology, Faculty of Science, University of South Bohemia in České Budějovice, 370 05 České Budějovice, Czech Republic; c Institute of Parasitology, Biology Centre of the Czech Academy of Sciences, 370 05 České Budějovice, Czech Republic; EMBL Grenoble, France

**Keywords:** serpins, Iripin-4, X-ray structure, native conformation, cleaved conformation, *Ixodes ricinus*

## Abstract

Iripin-4, one of the many salivary serpins from *Ixodes ricinus* ticks with an as-yet unexplained function, crystallized in two different structural conformations, namely the native partially relaxed state and the cleaved serpin. The native structure was solved at a resolution of 2.3 Å and the structure of the cleaved conformation was solved at 2.0 Å resolution. Furthermore, structural changes were observed when the reactive-centre loop transitioned from the native conformation to the cleaved conformation. In addition to this finding, it was confirmed that Glu341 represents a primary substrate-recognition site for the inhibitory mechanism. The presence of glutamate instead of the typical arginine in the P1 recognition site of all structurally characterized *I. ricinus* serpins (PDB entries 7b2t, 7pmu and 7ahp), except for the tyrosine in the P1 site of Iripin-2 (formerly IRS-2; PDB entry 3nda), would explain the absence of inhibition of the tested proteases that cleave their substrate after arginine. Further research on Iripin-4 should focus on functional analysis of this interesting serpin.

## Abstract 2

The crystal structures of two important conformations of a new serpin from *I. ricinus*, namely the partially stressed native and cleaved conformations, were solved at 2.3 and 2.0 Å resolution, respectively. The importance of the reactive-centre loop in protease inhibition was also confirmed.

## 1. Introduction

*Ixodes ricinus* (subclass Acari, order Ixodidae) is one of the most important European vectors, transmitting many pathogens, and is therefore also of veterinary and medical importance. This hard tick species attacks many cold- and warm-blooded vertebrate hosts, including humans. The two most commonly reported tick-borne infections in humans are Lyme disease caused by *Borrelia burgdorferi sensu lato* and the Western European subtype of tick-borne encephalitis virus, of which *I. ricinus* is the main vector in Europe (Rizzoli *et al.*, 2011 ▸; Madison-Antenucci *et al.*, 2020 ▸; Boulanger *et al.*, 2019 ▸).

As *I. ricinus* is widespread in Europe, efforts have been made to reduce tick abundance and thus reduce the risk of possible infection. One way to reduce tick numbers is to use acaricides, but ticks can develop resistance to these products in the long term. Another strategy is to use repellents, which often contain toxic substances that can cause environmental contamination, which has become a serious problem. Moreover, repellents provide only short-term protection (Rosario-Cruz *et al.*, 2009 ▸). A biological approach to tick control, such as vaccinating the host, appears to be the most viable option. This approach can induce protective host immunity, leading to host rejection of the tick. Tick saliva contains many important substances that could be crucial for developing a vaccine to block transmission of these substances. Serine protease inhibitors (serpins) have been tested in several studies, and recombinant serpins from several tick species have been described as candidates for vaccine design (de la Fuente *et al.*, 2016 ▸). For example, an *I. ricinus* serpin called Iris induced protective immunity against nymphs and adults fed on vaccinated rabbits but not against nymphs fed on mice (Prevot *et al.*, 2007 ▸; Meekins *et al.*, 2017 ▸).

With more than 6000 members, serpins are among the largest families of protease inhibitors. They are widespread in all organisms and function in many physiological processes (Silverman *et al.*, 2010 ▸; Spence *et al.*, 2021 ▸). Serpins differ from other groups of protease inhibitors mainly in their unique mode of inhibition. To inhibit the target protease, serpins undergo a fundamental conformational change that leads to inactivation of both the protease and the serpin itself. For this reason, serpins are referred to as ‘one use only’ or ‘suicide’ inhibitors (Whisstock *et al.*, 1998 ▸; Dunstone & Whisstock, 2011 ▸). Many serpins have functions other than those implied by their name. There is ample evidence that some serpins, such as the viral CrmA and plant serpin 1, can inhibit cysteine proteases in addition to serine proteases (Komiyama *et al.*, 1994 ▸; Vercammen *et al.*, 2006 ▸). The serpin family also contains non-inhibitory members with roles in, for example, hormone transport, protein folding, blood-pressure regulation, chromatin condensation and tumour progression (Kaiserman *et al.*, 2006 ▸).

The main structural feature of all serpins is a conserved tertiary structure consisting of a core domain of ∼350 amino acids. Thus, the secondary structure of serpins consists of three β-sheets (β-sheets A, B and C), at least eight α-helices (A–H) and a reactive-centre loop (RCL) (Gettins, 2002 ▸). The amino acids in the RCL, particularly the residues at the P1 and P1′ positions, determine the protease selectivity of serpins by mimicking the substrate of the target protease (Sanrattana *et al.*, 2019 ▸). Numerous crystallographic studies have provided a large amount of structural data on possible conformations of serpins that may be independent of the inhibition process. The conformations can be divided into a series of monomeric serpin structures (native fully stressed state, native partially relaxed state, latent conformation, abnormal δ-conformation and cleaved conformation) and protease-inhibited complexes (Michaelis–Menten complex and covalent complex) (Dunstone & Whisstock, 2011 ▸).

The mechanism of protease inhibition has been well explained, as all inhibitory serpins undergo a similar molecular process that is required for protease inhibition. This process begins with recognition of the exposed RCL of the native metastable serpin by the protease as a ‘substrate’ and the formation of a Michaelis–Menten complex. Subsequently, the RCL is cleaved by the protease between the P1 and P1′ sites, resulting in binding of the protease to the RCL; the protease is then transported to the other side of the serpin by the RCL. This transition of the serpin to the more thermally stable conformation is related to insertion of the RCL into β-sheet A as an additional β-strand and simultaneous inactivation of the serpin and the protease by the formation of a covalent complex. If the reaction does not proceed sufficiently rapidly, the process results in an inactivated serpin in its most thermally stable cleaved conformation and a released active protease (Dunstone & Whisstock, 2011 ▸; Huntington & Carrell, 2001 ▸; Huntington, 2011 ▸). The inhibition process represents a flexible structural change that can make serpins vulnerable to mutations. Such mutations can lead to pathological conditions called serpinopathies, which manifest as thrombosis, cirrhosis, emphysema, immune hypersensitivity and other diseases that result from serpin dysfunction (Whisstock *et al.*, 2010 ▸).

To fully understand tick serpins and their functions, it is necessary to uncover structural information about individual serpins and their complexes with proteases. This may represent an essential starting point for the design and development of new drugs. Therefore, X-ray crystallography was used to solve the structure of the *I. ricinus* serpin Iripin-4 in its original native conformation; the cleaved conformation represents the resulting failed inhibition.

## 2. Materials and methods

### 2.1. Protein cloning, expression and purification

A full-length cDNA sequence of the gene that encodes Iripin-4 was obtained from the salivary glands of female *I. ricinus* ticks fed on guinea pigs by reverse transcription using the Transcriptor First Strand cDNA Synthesis Kit (Roche Applied Science, Penzberg, Germany) during a salivary-gland transcriptome project (Schwarz *et al.*, 2013 ▸). Iripin-4 was amplified using the primers shown in Supplementary Table S1, cloned into a Champion pET-SUMO expression vector (Life Technologies, Carlsbad, California, USA) using NEBuilder HiFi DNA Assembly Master Mix (New England Biolabs, Ipswich, Massachusetts, USA) and transformed into *Escherichia coli* Rosetta 2 (DE3) pLysS competent cells (Novagen, Merck Life Science, Darmstadt, Germany). An overnight culture (10 ml l^−1^) was inoculated into autoinduction TB medium (100 µg ml^−1^ kanamycin, 34 µg ml^−1^ chloramphenicol) and incubated for 24 h at 25°C. Bacterial cells were then harvested and disrupted with a cell disruptor. Soluble SUMO-tagged Iripin-4 was purified using a HisTrap FF column (GE Healthcare, Chicago, Illinois, USA) and eluted with 200 m*M* imidazole. After the first purification, the His and SUMO tags were cleaved using SUMO protease [1:100(*w*/*w*)] overnight at laboratory temperature. Samples were then reloaded onto a HisTrap column to separate the tags from native serpin. This step was followed by ion-exchange chromatography using a HiTrap Q HP column (GE Healthcare), which ensured sufficient protein purity. The final protein concentration was 1.17 mg ml^−1^ in 20 m*M* Tris pH 7.4, 20 m*M* NaCl and the protein was stored at −80°C.

### 2.2. Analysis of complex formation of Iripin-4 with proteases

The formation of covalent complexes of Iripin-4 was performed as reported previously (Chmelar *et al.*, 2011 ▸). Selected serine proteases (trypsin, thrombin, fXa, APC, fXIa, plasmin and elastase) were incubated with equimolar amounts of Iripin-4 for 1 h at laboratory temperature (final concentration of 1 µ*M*). Serpin–protease complex formation was analysed on reducing SDS–PAGE using 12% gels followed by Coomassie staining. The second-order inhibition rate constants for native Iripin-4 against recombinant human granzyme B were then defined as described in Chmelar *et al.* (2011 ▸). In order to detect substrate hydrolysis while avoiding saturation of the reaction, the concentration used was chosen based on the biochemical properties of granzyme B. Thus, one unit of granzyme B hydrolyzes 1 pmol min^−1^ Ac-IEPD-pNA substrate (200 µ*M*) at 37°C. The assay was performed in triplicate at 37°C and stopped at each time point by addition of the aforementioned substrate. Prior to substrate addition, Iripin-4 was pre-incubated with granzyme B for 0–14 min at a protease concentration of 1 U µl^−1^ (0.2 U µl^−1^ final concentration). The assay was measured at five different concentrations of Iripin-4 and plotted against the serpin concentration. The slope of the line of best fit provided an estimate of the second-order rate constant. An appropriate buffer consisting of 20 m*M* Tris pH 7.4, 150 m*M* NaCl, 0.01% Triton X-100 was used. The rate of substrate hydrolysis was measured using a plate reader (BioTek Synergy H1 Plate Reader, USA; absorbance at 405 nm) in a 96-well plate.

### 2.3. Protein crystallization, X-ray data collection and processing

A suitable protein concentration for crystallization screening was determined using the Pre-Crystallization Test (PCT; Hampton Research, Aliso Viejo, California, USA). Crystallization experiments were performed using the sitting-drop vapour-diffusion technique in 96-well Swissci MRC 2-drop or 3-drop crystallization plates (Molecular Dimensions, Newmarket, Suffolk, United Kingdom). Initial screening of crystallization conditions and further crystallization experiments were carried out using an OryxNano crystallization robot (Douglas Instruments, Hungerford, United Kingdom). Commercially available crystallization kits (Crystal Screen, PEGRx, PEG/Ion and Index from Hampton Research and JBScreen JCSG++ from Jena Bioscience) were used to screen for crystallization conditions. Protein solution:well solution ratios of 1:0.5 and 1:1 (0.4:0.2 µl and 0.4:0.4 µl) were used with protein concentrations of 1.17 and 2.3 mg ml^−1^ and the drop solution was equilibrated against 50 µl reservoir solution at 4°C. Suitable crystallization conditions were identified as JBScreen JCSG++ conditions H8 [25%(*w*/*v*) PEG 3350, 0.1 *M* bis-Tris pH 5.5, 0.2 *M* sodium chloride] and H10 [25%(*w*/*v*) PEG 3350, 0.1 *M* bis-Tris pH 5.5, 0.2 *M* ammonium acetate]. A simple 2D gradient was used for these conditions to optimize the crystal quality in a larger volume (0.5:0.5 µl).

For data collection, both native and cleaved Iripin-4 crystals were mounted directly from the crystallization drop and flash-cooled in liquid nitrogen without further cryoprotection prior to measurements. Diffraction data were collected on BL14.1 at the BESSY II electron-storage ring operated by Helmholtz-Zentrum Berlin (Mueller *et al.*, 2015 ▸). Data for all crystals were processed using *XDS* (Kabsch, 2010 ▸) with the *XDSAPP* graphical user interface (Sparta *et al.*, 2016 ▸). The solvent content of the crystal was analysed with *MATTHEWS_COEF* from the *CCP*4 suite (Winn *et al.*, 2011 ▸) Data-collection statistics for both data sets are summarized in Table 1 ▸.

### 2.4. Structure determination and refinement

The structures of Iripin-4 were solved using the *CCP*4 suite (Winn *et al.*, 2011 ▸). The *MOLREP* (Vagin & Teplyakov, 2010 ▸) molecular-replacement method using *MrBUMP*, an automated pipeline for molecular replacement (Krissinel *et al.*, 2018 ▸), was used for the cleaved conformation, and the structure of Iripin-2 (PDB entry 3nda; Chmelar *et al.*, 2011 ▸), with 56.5% sequence identity, generated by an automated model search using the *BALBES* molecular-replacement pipeline (Long *et al.*, 2008 ▸) was used for the native conformation. The resolution cutoff for native Iripin-4 was 2.3 Å due to the presence of two ice rings at 2.25 and 1.95 Å resolution. The structures were refined using *REFMAC*5 (Murshudov *et al.*, 2011 ▸) from the *CCP*4 package (Winn *et al.*, 2011 ▸) and were manually rebuilt in *Coot* (Emsley *et al.*, 2010 ▸) based on evaluation of the electron-density peaks. Improvement during refinement was monitored by structure validation throughout the refinement process. Water molecules were added to the model using the *REFMAC*5 interface. The accepted solvent molecules had hydrogen-bond contacts with permissible geometry in the range 2.5–3.5 Å to protein atoms or to existing solvent. At this point, residues and water molecules with two possible conformations were included, and their alternative conformations were added for further refinement. In the final refinement steps, the Ni^2+^ ion (native structure) and four chloride ions (cleaved structure) were incorporated into the respective (2*F*_o_ − *F*_c_) and (*F*_o_ − *F*_c_) electron-density maps using coordinates from the ligand data bank in *Coot* (Emsley *et al.*, 2010 ▸). The *MolProbity* server (Chen *et al.*, 2010 ▸) and the wwPDB OneDep validation system (Young *et al.*, 2017 ▸) were used for final qualitative model validation. All figures were created by *PyMOL* (Schrödinger) or *CCP*4*mg* (Mc­Nicholas *et al.*, 2011 ▸). To visualize the cleaved conformation, chain *C* was used. The refinement statistics are summarized in Table 1 ▸.

## 3. Results

### 3.1. Recombinant Iripin-4 production and purification

The Iripin-4 gene transcript amplified from tick cDNA showed three amino-acid substitutions, H78Q, G155E and G307D, compared with the deposited Iripin-4 sequence (GADI01002650; Supplementary Fig. S1) used for primer design. This is not an artefact but is most likely to be the result of high inter-tick variability in the genes expressed in salivary glands, as reported in Kotál *et al.* (2021 ▸). The resulting protein has no substitutions in the RCL or the hinge region, and therefore should retain the same inhibitory function and specificity. The final yield of Iripin-4 was 17 mg (∼2 mg per litre of medium), which is a sufficient concentration for further experiments (Supplementary Fig. S2). Using the *NetNGlyc* 1.0 and *NetOGlyc* 4.0 servers, the amino-acid sequence of Iripin-4 was estimated to contain two potential N-glycosylation sites [N-*X*-(S/T)] and four potential O-glycosylation sites (Supplementary Table S2).

### 3.2. Complex formation of Iripin-4 with proteases

**Figure 1:**
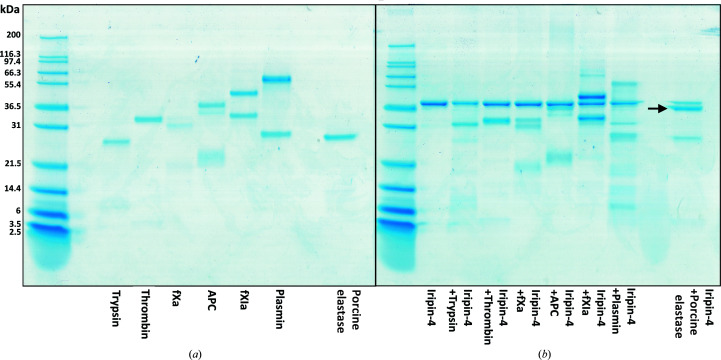
Analysis of heat-stable complex formation between Iripin-4 and tested proteases. No complex formation was observed. There is visible cleavage of Iripin-4 by porcine elastase, as shown by a black arrow. Proteins were resolved on 12% NuPAGE Bis-Tris gels and visualized by Coomassie staining.

**Figure 2:**
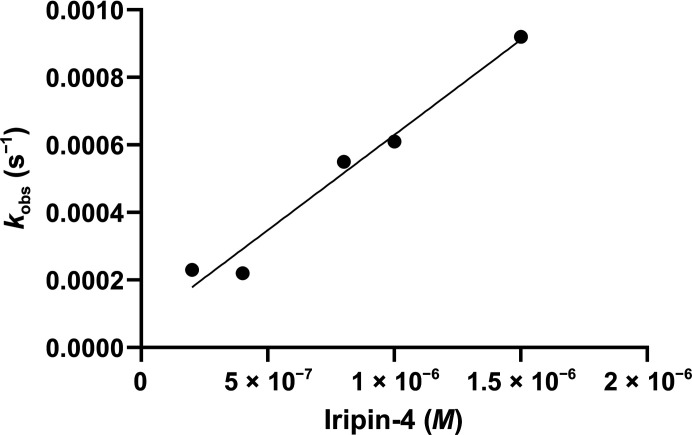
The relationship between Iripin-4 concentration and *k*_obs_, the observed second-order rate constant for the binding of human granzyme B to Iripin-4. *k*_obs_ was determined using a *para*-nitroanilide substrate (Ac-IEPD-pNA).

Iripin-4 is not a suitable inhibitor of the selected serine proteases. No complex formation was observed between Iripin-4 and any of the tested serine proteases. In addition, Iripin-4 acted as a substrate for porcine elastase, as can be observed as a double band on a NuPAGE gel corresponding to native and cleaved serpin (Fig. 1 ▸). For this reason the second-order rate constant for Iripin-4 inhibition of granzyme B was determined (Fig. 2 ▸). Granzyme B is a component of cytolytic granules produced by natural killer and cytotoxic T lymphocytes. Granzyme B serves as a mediator of inflammation, controls the coagulation cascade (the ability to trigger inflammatory cytokines) and has proteolytic activity that is crucial for programmed cell death (Garzón-Tituaña *et al.*, 2020 ▸). Iripin-4 protects cells by inhibiting granzyme B, and is also partly responsible for prolonging the attachment of the tick to the host. Mutants of Iripin-4 show similar but insignificant activity towards native Iripin-4 (data not shown).

### 3.3. Crystallization of Iripin-4 conformations

Based on initial screening, several successful crystallization conditions were found at both protein concentrations. Preliminary data collection indicated that optimization of crystallization was required, and therefore a protein concentration of 1.17 mg ml^−1^ with a larger drop volume (2 µl) was used for further optimization. Crystals of native Iripin-4 grew under conditions consisting of 25%(*w*/*v*) PEG 3350, 0.1 *M* bis-Tris pH 5.5, 0.2 *M* ammonium acetate (Supplementary Fig. S3). The difference in the crystallization conditions for the cleaved conformation was the presence of 0.2 *M* sodium chloride instead of 0.2 *M* ammonium acetate as used in the crystallization of the native serpin (Supplementary Fig. S4). One of the most likely reasons for obtaining crystals of both conformations was the storage time of the protein sample. In the case of native Iripin-4 the protein sample was freshly obtained, whereas the crystals of cleaved Iripin-4 were formed from a protein sample that had been stored at −20°C for six months before optimization was initiated.

### 3.4. The partially relaxed state of the native conformation

The structure of native Iripin-4 was solved at 2.3 Å resolution and the crystal belonged to space group *P*3_1_21 (No. 152), with unit-cell parameters *a* = 77.78, *b* = 77.78, *c* = 109.49 Å, α = 90.0, β = 90.0, γ = 120.0°. The crystal contained one molecule in the asymmetric unit with 51.33% solvent content and a Matthews coefficient of 2.53 Å^3^ Da^−1^. Detailed refinement statistics are given in Table 1 ▸.

**Figure 3:**
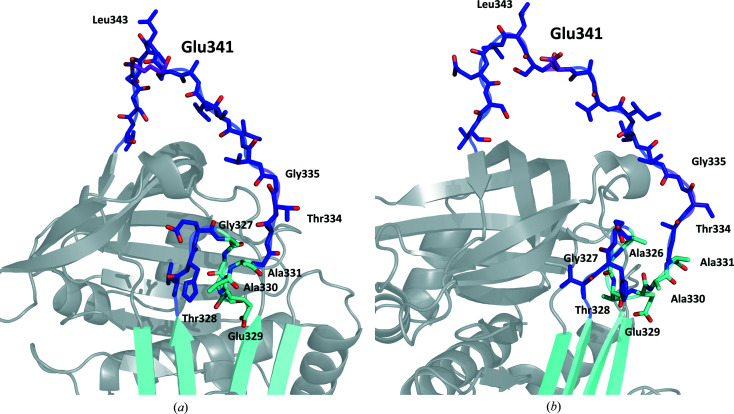
The RCL of native Iripin-4. (*a*) Front view of the RCL (blue) showing the inserted residues into β-sheet A (cyan) and highly flexible residues of the hinge region of the RCL (purple). (*b*) View rotated by 50°.

**Figure 4:**
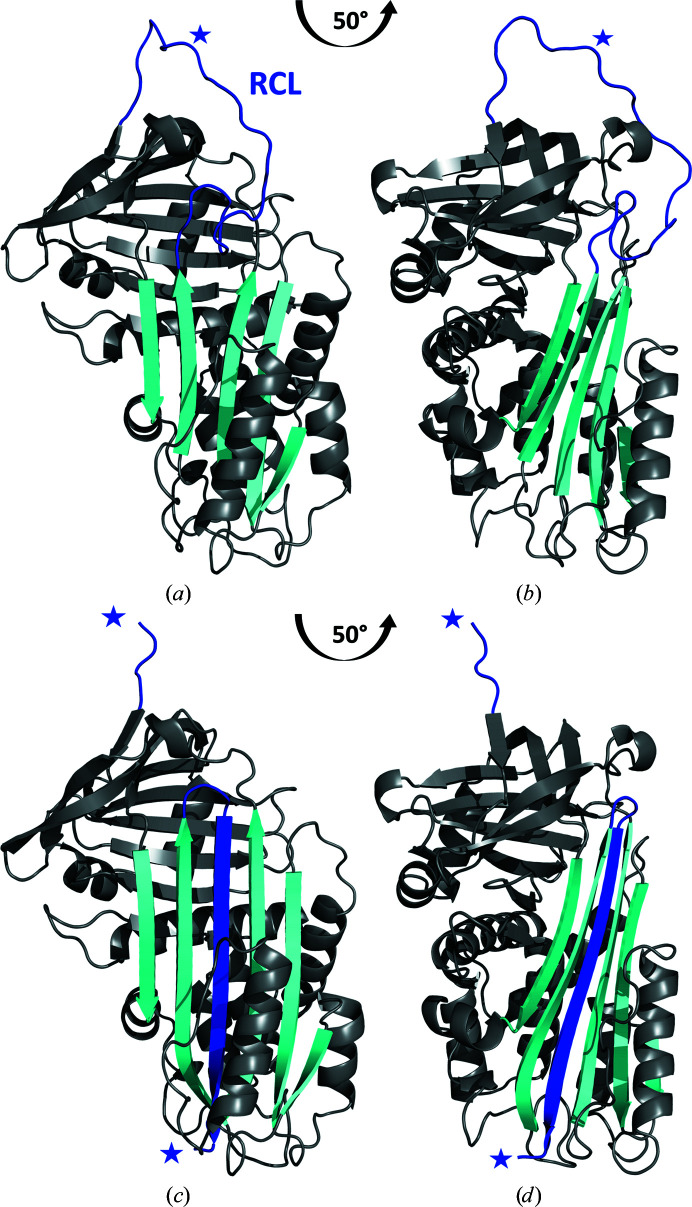
Crystal structures of Iripin-4. (*a*) Native Iripin-4; the position of the RCL partially inserted into the breach region of β-sheet A is highlighted. (*b*) Side view of the native conformation. (*c*) Cleaved Iripin-4 displaying an additional β-strand in β-sheet A as a result of RCL insertion. (*d*) Side view of the cleaved conformation. Both crystal structures are displayed as cartoons; β-sheet A is in cyan and the RCL is in blue. The position of the P1 cleavage site is marked by a blue asterisk.

The overall structure of Iripin-4 consists of all 376 residues with a typical fold of native serpin secondary structure with the RCL exposed on top of β-sheet C as a protease ‘bait (substrate)’ (Fig. 3 ▸; Huntington, 2011 ▸). The structure consists of nine α-helices and three β-sheets (A, B and C) arranged sequentially in the order αA–β1–αB–αC–αD–β2–αE–β3–αF–β4–β5–β6–β7–β8–αG–αH–β9–β10–αI–β11–β12–β13–β14 (Fig. 4 ▸ *b*). β-Sheet A consists of five β-strands, β-sheet B of five β-strands and β-sheet C of four β-strands. The crystal structure has a tentatively assigned nickel cation (Ni^2+^) located between two molecules of Iripin-4 with crystallographic symmetry that interacts with His253 and Asp257 of both molecules (Supplementary Fig. S5). The presence of the Ni^2+^ ion, which was the most logical choice, is probably due to release of this ion during affinity purification.

The crystal structure of Iripin-4 shows the typical partially relaxed state of native serpins, in which the RCL, which is located between β-sheet A and β-sheet C, is partially inserted into the breach region (Fig. 4 ▸ *a*; the top part of β-sheet A; Whisstock & Bottomley). In contrast, the remaining part of the RCL is exposed (Fig. 4 ▸ *b*). This state is likely to be the result of a crystallization process that trapped the serpin in one of two possible states, given the high flexibility of the RCL and the dynamic equilibrium between the partially inserted and the fully exposed native state. The six amino acids Ala326, Gly327, Thr328, Glu329, Ala330 and Ala331 are inserted between the third β-strand (s3A) and the fifth β-strand (s5A) of β-sheet A (Fig. 3 ▸). Three amino acids in the exposed part of the RCL, namely Thr334 and Gly335, which are part of the hinge region, and Leu343, were modelled into the structure despite the lower electron density observed here, indicating high flexibility of these two regions of the RCL (Fig. 3 ▸).

### 3.5. Structural transition to the cleaved conformation

The crystal of cleaved Iripin-4 diffracted to 2.00 Å resolution and belonged to space group *P*21 (No. 4), with unit-cell parameters *a* = 65.70, *b* = 138.41, *c* = 80.22 Å, α = 90.0, β = 107.7, γ = 90.0°. The crystal contains four molecules in the asymmetric unit, with a solvent content of 40.65% and a Matthews coefficient of 2.07 Å^3^ Da^−1^. Detailed refinement statistics are given in Table 1 ▸.

**Figure 5:**
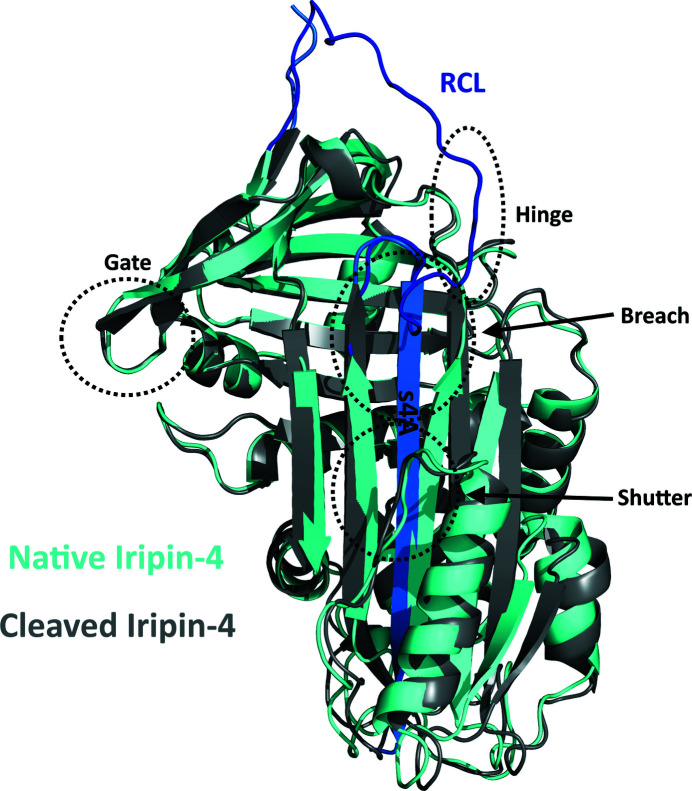
Structural superposition of Iripin-4 conformations. The native Iripin-4 (cyan) with an exposed RCL (blue) is aligned with cleaved Iripin-4 (grey) containing a new additional β-strand (marine) labelled s4A. The three regions responsible for insertion of the RCL into β-sheet A are marked in circles (Khan *et al.*, 2011 ▸). The hinge region is marked with an oval.

The structure of the cleaved conformation consists of a typical mixed α–β secondary serpin structure with an N-terminal helical region and a C-terminal β-sheet fold. The overall structure consists of nine α-helices (A–I) and three β-sheets arranged sequentially in the order αA–β1–αB–αC–αD–β2–αE–β3–αF–β4–β5–β6–β7–β8–αG–αH–β9–β10–αI–β11–β12–β13–β14–β15 (Fig. 4 ▸ *c*). The additional β12 strand results from the insertion of an RCL between the two β-strands of β-sheet A to form an additional strand called s4A; more precisely, it is located between the β11 strand (s5A) and the β4 strand (s3A) (Fig. 4 ▸ *d*). A chloride ion (Cl^−^) is present in the model at the same position in all chains and interacts with Leu215 in the gate region. The gate region (Fig. 5 ▸) is responsible for stabilizing the RCL and preventing its premature insertion into β-sheet A (Irving *et al.*, 2015 ▸).

The four molecules in the final model differ in the number of modelled amino acids near the P1 site. Chains *A* and *D* contain 373 residues, with amino acids Ser342, Leu343 and Val344 missing, compared with chain *B*, which lacks Ser342 and Leu343 and consists of 374 residues, and chain *C*, in which only Ser342 is missing and which consists of 375 residues (out of a total of 376 residues). The missing residues were observed as an absence of electron density after the P1 residue Glu341 located at the end of the inserted RCL, now called s4A (Fig. 5 ▸). Alignment of the chains using *PyMOL* (Schrödinger) showed minor differences, which were confirmed by r.m.s.d. calculations between chain *C* (3014 atoms) and the other three chains: chain *A*, r.m.s.d. of 0.181 Å (2351 atoms); chain *B*, r.m.s.d. of 0.270 Å (2424 atoms); chain *D*, r.m.s.d. of 0.271 Å (2367 atoms).

### 3.6. Structural differences in native and cleaved Iripin-4

Structural comparison of the native and cleaved conformations of Iripin-4 shows that the β-strands in β-sheet A, together with α-helix D, α-helix E, α-helix F and α-helix I, shift to make space for the inserted RCL (Fig. 5 ▸). Since the cleaved conformation of serpin is structurally most related to the structure of the covalent complex between the serpin and the protease, it can be concluded that these structural changes will be the same or at least similar to the changes required for formation of the covalent complex. These observations also show how a significant conformational change is required for transition from the native to the cleaved state and also for protease inhibition. In addition to the structural changes in the shutter and hinge regions, a shift of the gate region, the loop part of β-sheet C between the s3C and s4C strands, is evident (Fig. 5 ▸).

**Figure 6:**
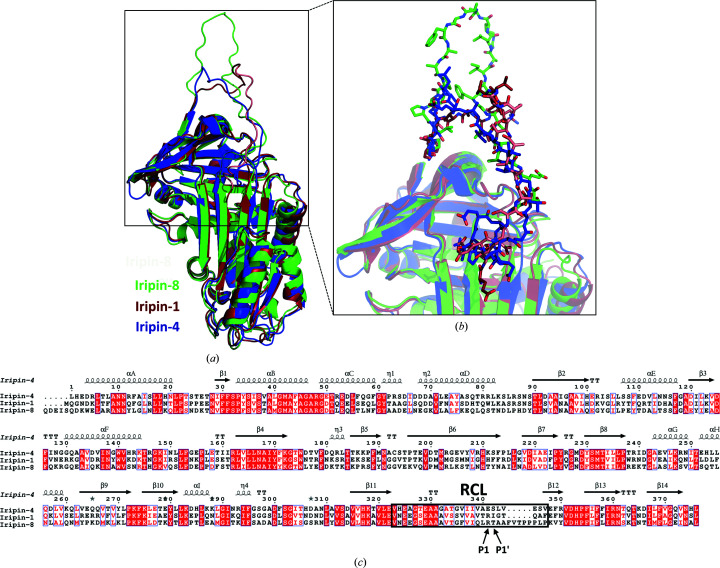
Superposition and alignment of *I. ricinus* native serpin conformations. (*a*) Superposition of the Iripin-4 (PDB entry 7zbf), Iripin-1 (PDB entry 7qtz; Chlastáková *et al.*, 2023 ▸) and Iripin-8 (PDB entry 7pmu) crystal structures displayed as cartoons. The structure models are differentiated as follows: Iripin-4 is in blue, Iripin-1 is in red and Iripin-8 is in green. The structure of Iripin-1 was modified because of the absence of residues in its RCL, which are coloured salmon for emphasis. (*b*) A closer view of the RCL residues displayed as sticks; the colours are as described previously. (*c*) Structure-based sequence alignment of *I. ricinus* native serpins. The secondary-structure elements are depicted as arrows for β-strands and spirals for α-helices. The RCL is highlighted in a black box and the P1 and P1′ residues are marked according to the nomenclature of Schechter & Berger (1967 ▸).

The aforementioned breach, shutter, hinge and gate regions are involved in the incorporation of the RCL, specifically the hinge region of the RCL, into β-sheet A through coordinated movements in this complex process (Khan *et al.*, 2011 ▸; Pearce *et al.*, 2007 ▸). This process is referred to as the S-to-R transition, which involves a transition from a native stressed state to a more relaxed cleaved state with greater thermal stability (Whisstock *et al.*, 2000 ▸). Comparison of the native and cleaved structures of Iripin-4 (Fig. 5 ▸) shows significant movement in the breach and shutter regions. The breach region, which is the starting point in the inhibitory mechanism, was slightly open compared with other solved structures of native serpins from *I. ricinus* (Fig. 6 ▸) and thus is more flexible than the shutter region, which is responsible for controlling or preventing undesired conformational change. The shutter region has more than 85% conserved residues and must open to incorporate the RCL, which helps to stabilize this transition (Irving *et al.*, 2000 ▸). The polar contacts between s3A and s5A, more precisely Glu316–Asn167, Lys318–Asn167 and Thr319–Ile169, were disrupted by the insertion of the RCL compared with the cleaved structure with the additional β-s4A strand. For proper inhibition of the RCL by the protease, the transition must occur outside the gate region (Khan *et al.*, 2011 ▸) and result in cleavage of the serpin instead of the formation of a covalent complex with the protease.

## 4. Discussion

Iripin-4 does not show inhibition by the typical mechanism of enzyme inhibition, *i.e.* by forming a covalent complex with the tested target protease. The most prevalent residue in the P1 site differs from the arginine residue found in other *I. ricinus* serpins with known structures, namely Iripin-5, Iripin-8 and Iripin-3 (Arg342 in PDB entry 7b2t, Arg364 in PDB entry 7pmu and Arg357 in PDB entry 7ahp, respectively). The other known *I. ricinus* serpins contain Glu341 (Iripin-4), Tyr341 (Iripin-2, formerly named IRS-2; PDB entry 3nda) or Met340 (Iris) at the P1 position (Supplementary Fig. S6; Chmelar *et al.*, 2011 ▸; Kotál *et al.*, 2021 ▸; Chlastáková *et al.*, 2021 ▸; Kascakova *et al.*, 2021 ▸; Prevot *et al.*, 2006 ▸). The first serpin characterized in *I. ricinus* saliva was the immunosuppressant Iris, which inhibits elastase-like proteases. Iris prevents blood clotting by inhibiting factor Xa and thrombin, delays fibrinolysis by inhibiting elastase and tPA, and inhibits the secretion of pro-inflammatory cytokines such as IFNγ and IL-6. Some of these activities are the result of several Iris exosites in the αA and αD areas (Leboulle *et al.*, 2002 ▸; Prevot *et al.*, 2006 ▸, 2009 ▸). Another well described serpin from the same tick species is Iripin-2, which targets chymotrypsin-like proteases. Iripin-2 inhibits the pro-inflammatory proteinases cathepsin G and mast-cell chymase. By inhibiting thrombin, Iripin-2 blocks platelet aggregation and has been shown to act as an immunomodulator by inhibiting IL-6 production via altering STAT3 signalling (Chmelar *et al.*, 2011 ▸; Páleníková *et al.*, 2015 ▸). Another serpin from *I. ricinus*, Iripin-3, inhibits trypsin-like proteases, with the highest rates of inhibition against kallikrein and matriptase, and has been shown to suppress inflammation and wound healing. Iripin-3 also suppresses the proliferation and differentiation of CD4^+^ T cells into a Th1 pro-inflammatory subpopulation, thus regulating adaptive and acquired immune responses (Chlastáková *et al.*, 2021 ▸). Iripin-5, the most abundant salivary serpin in *I. ricinus*, displays an anti-inflammatory role as it inhibits neutrophil migration and complement activity and suppresses the ability of macrophages to produce NO (Kascakova *et al.*, 2021 ▸). Iripin-8 is an inhibitor of multiple coagulation proteases and strongly inhibits the intrinsic pathway of coagulation. In addition, it inhibits complement-related erythrocyte lysis. Thus, Iripin-8 functions as an anti­coagulant and inhibitor of the complement system (Kotál *et al.*, 2021 ▸). The presence of a partially inserted RCL in Iripin-4 may have led to the observed low inhibition of proteases. This was observed for antithrombin, which had a P1 site that was inaccessible to the target protease in this state. However, it is likely that Iripin-4 targets granzyme B, but weakly. The glutamate at the P1 site indicated that the target is granzyme B. As more functional analyses are needed, exosite-mediated activity similar to Iris cannot be ruled out. Further studies should address the precise function of Iripin-4.

Over the years of crystallographic study of serpins, many structural conformations and states have been identified and the importance of certain structural regions such as the breach, shutter and hinge regions have been described (Dunstone & Whisstock, 2011 ▸). The only serpin conformation with inhibitory function is the native conformation, which is metastable and stressed (Pearce *et al.*, 2007 ▸). This type of conformation has been solved for Iripin-4 at 2.3 Å resolution and represents an active serpin ready to ‘trap’ the target protease. The breach region of native Iripin-4 differs from those of other solved native *I. ricinus* serpin structures (Fig. 6 ▸). This is primarily due to the fact that the native structures of Iripin-1 (PDB entry 7qtz; Chlastáková *et al.*, 2023 ▸) and Iripin-8 (PDB entry 7pmu; Kotál *et al.*, 2021 ▸) were solved in the fully stressed state, as opposed to Iripin-4, which was solved in the partially relaxed state (Fig. 6 ▸). The breach region, which is located at the top of the s3A and s5A β-strands of β-sheet A, is opened laterally mainly by moving s3A to the site to create space for partial insertion of the RCL. In contrast, Iripin-1 and Iripin-8 do not have this gap in the breach region (Fig. 6 ▸). The shutter region (the middle part of β-sheet A) of all three serpins is almost identical. Another region that is part of the RCL (P15–P9), the hinge region, differs structurally between the three serpins because this region is responsible for the mobility of the RCL and its insertion into β-sheet A (Hopkins *et al.*, 1993 ▸; Sanrattana *et al.*, 2019 ▸). The hinge regions also differ in sequence: while Iripin-4 has threonine at the P14 site and glycine at the P10 site, Iripin-1 and Iripin-8 have serine at the P14 site and alanine at the P10 site. However, this does not suggest any functional difference, as it has been shown that uncharged residues are suitable for loop insertion but do not affect the protease selectivity (Lawrence *et al.*, 2000 ▸). It has been suggested that the use of cofactors during crystallization can lead to a fully exposed RCL state by stabilizing the hinge region (Whisstock & Bottomley, 2006 ▸; Li *et al.*, 2004 ▸), but this has not been observed for the mentioned proteins. The most remarkable part of native *I. ricinus* serpins is the length of the RCL, specifically the Iripin-8 RCL, which is longer by five residues. Another difference is that Iripin-4 has a P1 glutamate in its recognition site, whereas Iripin-1 and Iripin-8 have an arginine. Glutamate residues located at P4′ and P5′ have been reported to increase the rate of serpin inhibition (Ibarra *et al.*, 2004 ▸). The r.m.s.d. between molecules was calculated using *PyMOL* (Schrödinger) and the results showed that the r.m.s.d. on alignment of Iripin-4 with the Iripin-1 model was 0.894 Å (2008 atoms) and that with Iripin-8 was 1.143 Å (1943 atoms). Iripin-8 inhibits serine proteases involved in coagulation and complement functions. These proteases include thrombin, factor Xa, factor XIa, factor VIIa, factor IXa, kallikrein and plasmin (Kotál *et al.*, 2021 ▸). In contrast, Iripin-4 shows no significant inhibition of the tested proteases.

**Figure 7:**
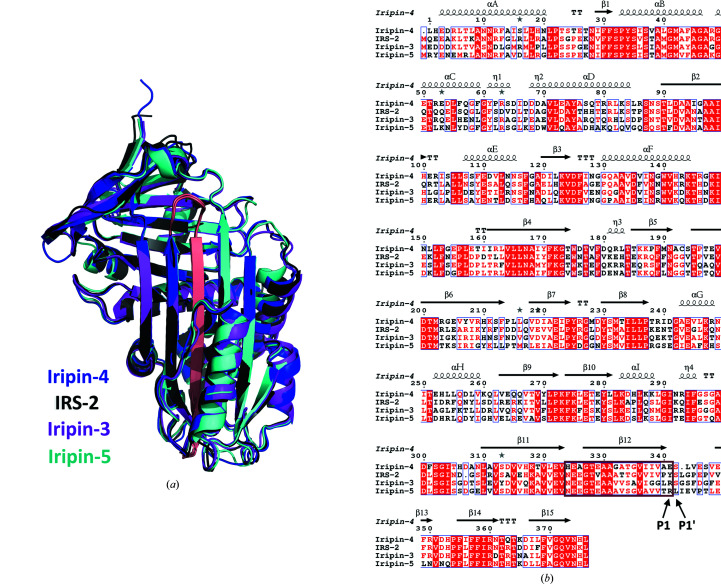
Superposition and alignment of *I. ricinus* cleaved serpin conformations. (*a*) Superposition of the Iripin-4 (PDB entry 7zas), Iripin-2 (PDB entry 3nda), Iripin-3 (PDB entry 7ahp) and Iripin-5 (PDB entry 7b2t) crystal structures displayed as cartoons. The structure models are differentiated as follows: Iripin-4 is in blue, Iripin-2 is in grey, Iripin-3 is in magenta and Iripin-5 is in cyan. The RCL of all serpins is coloured salmon. (*b*) Structure-based sequence alignment of *I. ricinus* cleaved serpins. The secondary-structure elements are marked with arrows for β-strands and spirals for α-helices. The inserted RCL is highlighted in the black box and the P1 and P1′ residues are marked according to the nomenclature of Schechter & Berger (1967 ▸).

Comparison of cleaved *I. ricinus* structures, specifically Iripin-2 (PDB entry 3nda; Kovářová *et al.*, 2010 ▸), Iripin-3 (PDB entry 7ahp; Chlastáková *et al.*, 2021 ▸) and Iripin-5 (PDB entry 7b2t; Prevot *et al.*, 2006 ▸), with the cleaved structure of Iripin-4 showed almost identical structural folding (Fig. 7 ▸). The results of alignment calculations showed that the r.m.s.d. on alignment of Iripin-4 with Iripin-2 is 0.750 Å (2212 atoms), that with Iripin-3 is 0.525 Å (2090 atoms) and that with Iripin-5 is 0.690 Å (2132 atoms). The differences are mainly in loop regions and mainly in the αD helix.

## 5. Conclusions

In conclusion, we have been able to define two structurally distinct steps in the likely mechanism of protease inhibition by Iripin-4. However, Iripin-4 was shown to inhibit only granzyme B protease, as no other protease tested was confirmed to be inhibited. Iripin-4 has also been shown to serve as a substrate for porcine elastase. The inhibition of granzyme B can be explained by the presence of Glu341 at the P1 site, which should indicate that Iripin-4 should be a potent inhibitor of this protease. However, Iripin-4 did not exhibit potent inhibition against granzyme B. Since the native structure was solved in a partially relaxed state, this cannot be ruled out as one of the reasons for the observed weak inhibition rate, although it may still be only an artefact of crystallization. In addition, it can be hypothesized that Iripin-4 may use a mechanism of inhibition other than the typical one or that some cofactors are required to increase the activity of this inhibitor. Further activity studies are needed to confirm the exact function of this serpin during the tick-feeding process.

## Supplementary Material

PDB reference: Iripin-4, cleaved, 7zas

PDB reference: native, 7zbf

Supplementary Methods, Supplementary Tables and Supplementary Figures. DOI: 10.1107/S2059798323002322/jv5014sup1.pdf

## Figures and Tables

**Table 1:** X-ray data-collection and refinement statistics Values in parentheses are for the highest resolution shell.

	Native Iripin-4	Cleaved Iripin-4
Data collection		
X-ray diffraction source	BL14.2, BESSY II, Germany	BL14.2, BESSY II, Germany
Wavelength (Å)	0.9184	0.9184
Temperature (K)	100	100
Detector	PILATUS 6M	PILATUS 6M
Crystal-to-detector distance (mm)	175.66	373.61
Rotation range per image (°)	0.10	0.10
Total rotation range (°)	270.00	360.00
Exposure time per image (s)	0.10	0.10
Resolution range (Å)	44.61–2.30 (2.44–2.30)	46.42–2.00 (2.12–2.00)
Space group	*P*3_1_21 [No. 152]	*P*2_1_ [No. 4]
Molecules in asymmetric unit	1	4
*a*, *b*, *c* (Å)	78.93, 78.93, 117.78	65.70, 138.41, 80.22
α, β, γ (°)	90.00, 90.00, 120.00	90.00, 107.70, 90.00
Mosaicity (°)	0.105	0.128
Total No. of reflections	289932 (43011)	634544 (98469)
No. of unique reflections	19373 (3085)	91690 (14692)
Multiplicity	14.97	6.92
Average *I*/σ(*I*)	22.47 (4.30)	9.10 (2.32)
Completeness (%)	99.70 (99.70)	99.40 (98.60)
CC_1/2_	99.90 (79.70)	99.70 (77.30)
*R*_meas_ † (%)	9.60 (62.40)	16.70 (85.30)
Overall *B* factor from Wilson plot (Å^2^)	39.20	23.00
Refinement		
Resolution range (Å)	44.62–2.30	46.42–2.00
No. of reflections in working set	18404	89590
Final *R* ‡/*R*_free_ § (%)	20.30/25.40	19.30/25.30
Mean *B* value (Å^2^)	39.80	28.00
No. of atoms in the asymmetric unit		
Protein	2962	11869
Ligand: chloride ion		4
Ligand: nickel ion	1	
Water	196	1276
Total	3168	13196
R.m.s. deviations		
Bond lengths (Å)	0.007	0.007
Angles (°)	1.444	1.455
Average *B* factors, overall (Å^2^)	42.0	28.0
Ramachandran plot		
Most favoured (%)	95.99	98.17
Allowed (%)	4.01	1.83
*MolProbity* score	1.46 [99th percentile¶]	1.27 [99th percentile¶]
PDB code	7zbf	7zas

† *R*_meas_ = 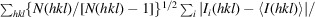 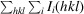 , where the average intensity 〈*I*(*hkl*)〉 is taken over all symmetry-equivalent measurements and *I*(*hkl*) is the measured intensity for any given reflection.

‡ *R* = 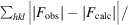 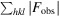 , where *F*_obs_ and *F*_calc_ are the observed and calculated structure factors, respectively.

§ *R*_free_ is equivalent to *R* but is calculated for 1.87% of the reflections chosen at random and omitted from the refinement process.

¶ The 100th percentile is the best among structures of comparable resolution; the 0th percentile is the worst.
