# Supplementary material for: Conformational transition of the Ixodes ricinus salivary serpin Iripin-4
Source: Acta Crystallogr D Struct Biol. 2023 Apr 24;79(Pt 5):409–19. doi: 10.1107/S2059798323002322 (PMC10167670; doi:10.1107/S2059798323002322)
Supplement: Supplementary file 1 [file d-79-00409-sup1.pdf]

STRUCTURAL
BIOLOGY

Volume 79 (2023)

Supporting information for article:

Conformational transition of the *Ixodes ricinus* salivary serpin Iripin-4

Barbora Kascakova, Jan Kotal, Petra Havlickova, Vera Vopatkova, Tatyana Prudnikova, Pavel Grinkevich, Michal Kuty, Jindrich Chmelar and Ivana Kuta Smatanova

S1. Cloning, Expression and Purification of Iripin-4 mutants

P1 site mutants (E341A and E341R) were prepared using Q5® Site-Directed Mutagenesis Kit (New England Biolabs Inc., Ipswich, MA, USA) according to the manufacturer's instructions. The primers used are listed in Supplementary Table S1. Both mutants were sequenced to confirm the P1 site substitution. The mutants were cloned into a pET-19b expression vector (Novagen, Merck Life Science, Darmstadt, Germany) and transformed into BL21(DE3) competent cells (New England Biolabs Inc., Ipswich, MA, USA). Overnight culture (20ml/l) was inoculated into 800 ml LB medium (100 µg*ml^{-1} ampicilin) and incubated for 8 h at 37°C. The bacterial cells were then harvested and disrupted using a cell disruptor. Soluble His-tagged Iripin-4 mutants were purified using a HisTrap HP column (GE Healthcare) and eluted with 200 mM imidazole. Samples containing Iripin-4 mutants were then loaded separately onto a HiTrap column (GE Healthcare) and subsequently onto Superdex 75 Increase 10/300 GL (GE Healthcare). The final concentrations of mutants were 0.28 mg/ml (E341A) and 0.40 mg/ml (E341R) in 20 mM Tris pH 7.4, 100 mM NaCl and the protein was stored at -80°C.

Table S1 List of primers used for amplification of Iripin-4 and both Iripin-4 mutants genes.

Amplicon	Forward primer 5‘ – 3‘	Reversed primer 5‘ – 3‘
**Iripin-4**	CACAGAGAACAGATTGGTGGACT CCACGAAGATAGACTGAC	GTCTCCTGAGTTCTAGAGTACTTTA TTAAAGATGATTGACCTGTCCC
**Iripin-4 Ala**	GGAGGTCCACgcaGCAGGCACCG	AGGACGGTCTTGTGGACC
**Iripin-4 Arg**	GGAGGTCCACagaGCAGGCACCG	AGGACGGTCTTGTGGACC

Table S2 Analysis of potential glycosylation sites using NetOGlyc - 4.0 and NetNGlyc - 1.0

**O-glycosylation sites**		**N-glycosylation sites**	
**position**	**potential**	**position**	**potential**
**79**	0.6335	**88** NSTL	0.7152
**145**	0.6004	**249** NITE	0.6120
**184**	0.5063		
**185**	0.5122		

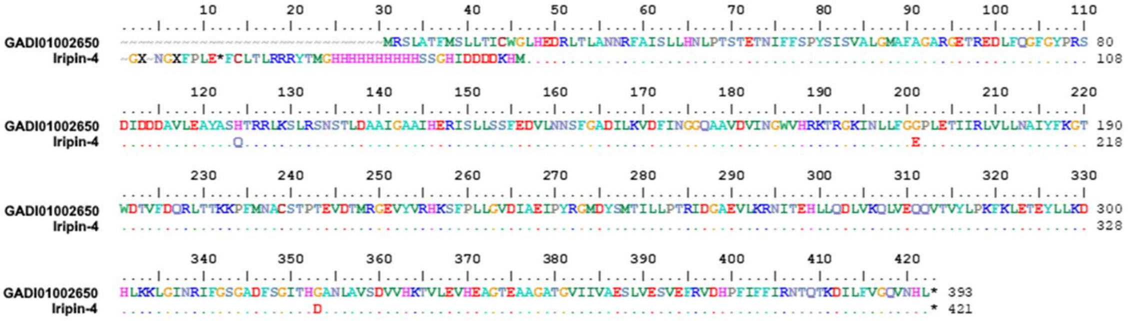

Figure S1 Comparison of cDNA sequence (GADI01002650) obtained from a salivary gland transcriptome project (Schwarz *et al*., 2013) with a sequence of Iripin-4.

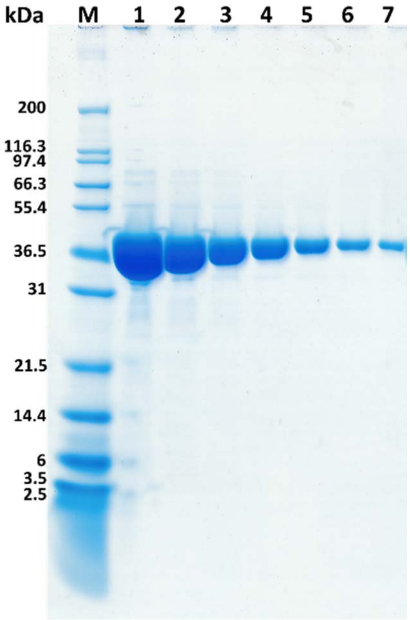

Figure S2 Iripin-4was analyzed by a reducing SDS-PAGE gel. M: Molecular weight marker, 1-7: Iripin-4 with a load of 50, 25, 12.5, 6.2, 3.1, 1.55, 0.8 µg per well.

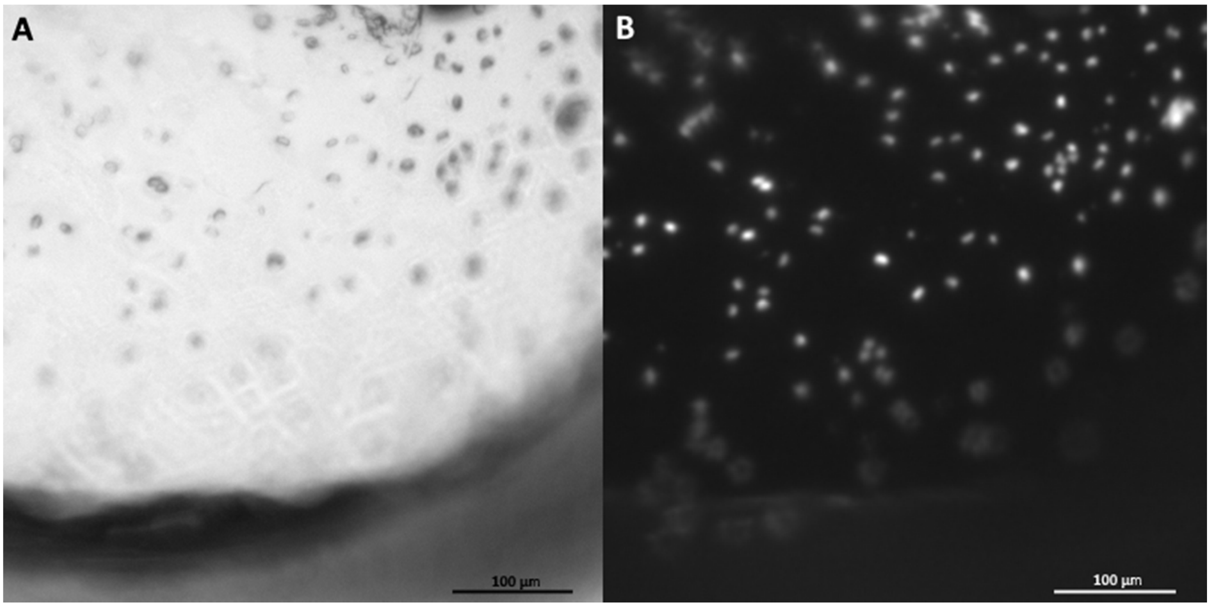

Figure S3 Crystals of native Iripin-4. (A) Crystals of protein grown in 25%(w/v) PEG 3350, 0.1 M Bis-Tris pH 5.5, 0.2 M ammonium acetate. (B) The same crystallization droplet is shown under UV light. (A) and (B) were taken using a JANSi UVEXm (SWISSCI, UK). The scale bar represents100 µm.

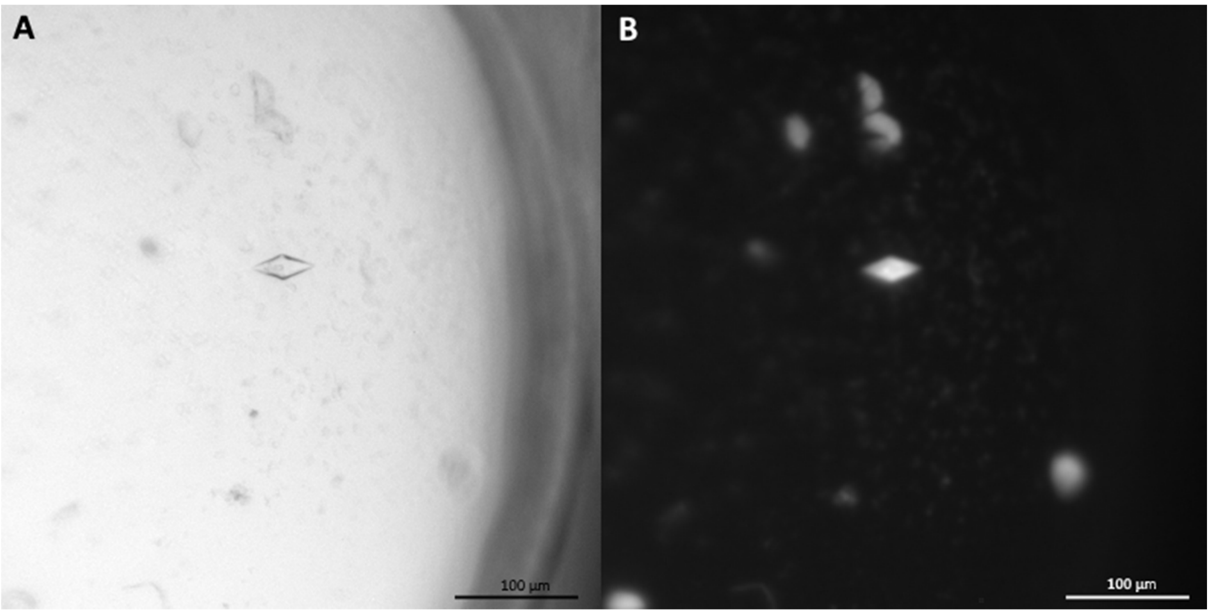

Figure S4 Crystals of cleaved Iripin-4. (A) Crystals of protein grown in 25%(w/v) PEG 3350, 0.1 M Bis-Tris pH 5.5, 0.2 M sodium chloride. (B) The same crystallization droplet is shown under UV light. (A) and (B) were taken using a JANSi UVEXm (SWISSCI, UK). The scale bar represents100 µm.

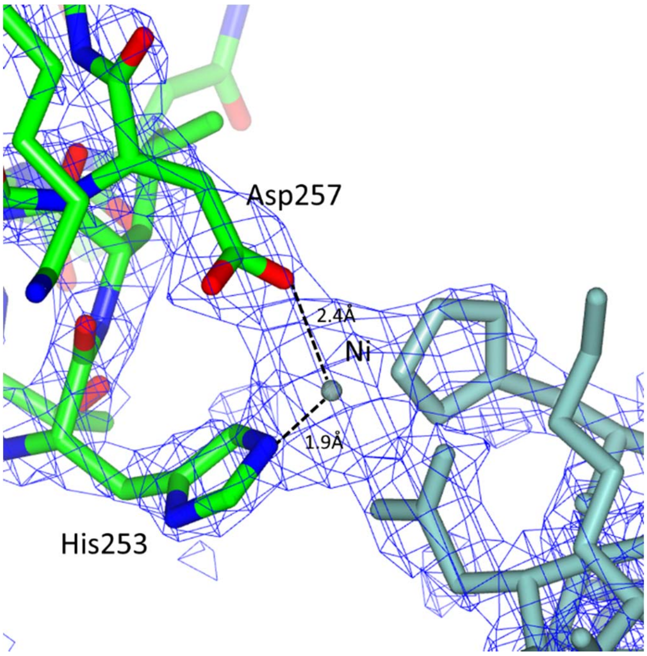

Figure S5 Visualization of the Nickel cation between two molecules of Iripin-4 in crystal. The Asp257 and His253 interacting with Ni2+are marked. The distances between interacting atoms are 1.9 Å for His253 Nε2 and Ni2+, and 2.4 Å for Asp257 Oδ2 and Ni2+

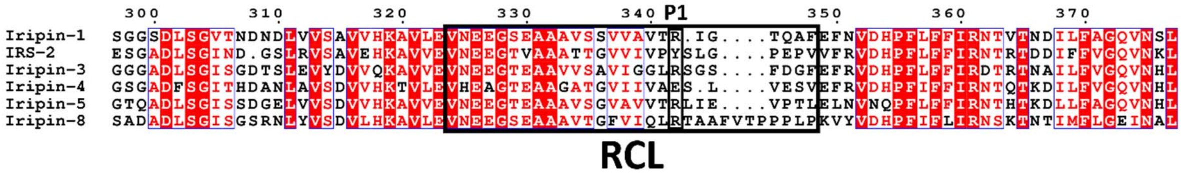

Figure S6 Comparison of RCL region of *I. ricinus* serpins. The RCL is in the black box with prompted P1 recognition site in the smaller black box.

Schwarz A, von Reumont BM, Erhart J, Chagas AC, Ribeiro JMC, Kotsyfakis M. (2013). *FASEB J* **27**:4745–4756.
